# Desflurane anesthesia shifts the circadian rhythm phase depending on the time of day of anesthesia

**DOI:** 10.1038/s41598-020-75434-6

**Published:** 2020-10-26

**Authors:** Ryo Imai, Hiroshi Makino, Takasumi Katoh, Tetsuro Kimura, Tadayoshi Kurita, Kazuya Hokamura, Kazuo Umemura, Yoshiki Nakajima

**Affiliations:** 1 Department of Anesthesiology, Hamamatsu University School of Medicine, 1-20-1 Handayama, Hamamatsu, Shizuoka 431-3192 Japan; 2 Department of Medical Education, Hamamatsu University School of Medicine, Hamamatsu, Japan; 3 Department of Pharmacology, Hamamatsu University School of Medicine, Hamamatsu, Japan

**Keywords:** Circadian rhythms and sleep, Circadian mechanisms

## Abstract

Desflurane is one of the most frequently used inhalational anesthetics in clinical practice. A circadian rhythm phase-shift after general anesthesia with sevoflurane or isoflurane has been reported in mice, but few studies have reported this effect with desflurane. In the present study, we examined the rest/activity rhythm of mice by counting the number of running wheel rotations, and we found that desflurane anesthesia caused a phase shift in the circadian rhythm that was dependent on the time of day of anesthesia. We also found that desflurane anesthesia altered the relative mRNA expression of four major clock genes (*Per2*, *Bmal*, *Clock*, and *Cry1*) in the suprachiasmatic nucleus (SCN). These results are important for elucidating the effects of desflurane on the SCN, which is the master clock for the mammalian circadian rhythm. Further studies on the relationship between anesthesia and circadian rhythm may lead to the prevention and treatment of postoperative complications related to circadian rhythms.

## Introduction

The circadian rhythm is an innate ability in almost all living things. In mammals, the circadian rhythm master clock is in the suprachiasmatic nucleus (SCN), and it controls the circadian rhythm of behavior, circulation, and the endocrine system^1^. Circadian rhythm disorders may cause various diseases^2,3^. Postoperative sleep disorder is one of the most frequent symptoms caused by abnormal circadian rhythms^4^. Postoperative delirium and cognitive decline may also be associated with an abnormal circadian rhythm^5^. These symptoms decrease the quality of life of postoperative patients and their families and are also risk factors for other fatal complications^6,7^. Therefore, elucidating the mechanism of postoperative circadian rhythm disorder is clinically important.

Sevoflurane, isoflurane, and desflurane are the most commonly used inhalational anesthetics in clinical practice. General anesthesia with sevoflurane or isoflurane affects the circadian rhythm by altering clock gene expression in the SCN^8,9^. Furthermore, previous reports suggested that the effect of sevoflurane and isoflurane anesthesia on the circadian rhythm might differ depending on the time of day of anesthesia^10,11^. However, the effects of desflurane general anesthesia on the circadian rhythm have not been reported. Desflurane has a significantly lower solubility in blood compared with other volatile anesthetics and is eliminated through exhalation without being metabolized. These characteristics contribute to the rapid onset and offset of desflurane anesthesia, and in fact, anesthesiologists often administer desflurane as general anesthesia to quickly wake patients after surgery. Based on these characteristics, desflurane might have a different effect on the circadian rhythm compared with other inhaled anesthetics^12^.

The circadian clock is an endogenous mechanism that controls 24-h physiological processes through a transcriptional feedback loop of clock genes. Recent studies have identified many important clock genes involved in regulating the circadian clock in mammals. Among them, *Period *(*Per*), *Bmal*, *Clock*, and *Cryptochrome *(*Cry*) are the most well-known and particularly important clock genes for the formation of the circadian rhythm. Entrainment of the circadian clock in mammals mainly depends on light stimuli, which are received by intrinsically photosensitive retinal ganglion cells (ipRGCs) and inputted into the SCN via the retinohypothalamic tract (RHT)^13^. Nonphotic entrainment in mammals has also been studied. Exercise, mealtime, social contact, and pharmacological compounds are known to have chronobiotic potential^14^. In recent studies, anesthetics have been investigated as drugs with chronobiotic potential. Volatile anesthetics, such as sevoflurane, epigenetically suppress *Period2* transcription by inhibiting the acetylation of the *Period2* promoter, thereby causing a phase shift in the circadian rhythm of mice^15^. In addition, the recovery time from sevoflurane and isoflurane anesthesia is different in the circadian phase, and it involves the locus coeruleus noradrenergic system^16^. However, there is limited information regarding the effects of desflurane on the circadian rhythm and clock gene expression. In addition, whether the effects of desflurane are dependent on the time of day of anesthesia is poorly understood.

In this study, we quantitatively evaluated the effect of desflurane anesthesia on the circadian rhythm by monitoring the rest/activity rhythm in mice and examining its dependence on the time of day of anesthesia. In addition, to determine the effect of desflurane anesthesia on the master clock, the expression of four major clock genes in the SCN was measured.

## Materials and methods

### Animal preparation

Experiments were conducted in accordance with guidelines approved by the Animal Care Committee of the Hamamatsu University School of Medicine Animal Care Facility. Adult (6–8 weeks old, mean body weight ± SD of 20.9 ± 1.4 g) male C57BL/6 J mice (SLC, Hamamatsu, Japan) were used for all experiments.

Mice were housed for at least 10 days to allow adaptation to the light:dark conditions (LD), which consisted of a 12-h light (from zeitgeber time [ZT]0 to ZT12) and 12-h dark (from ZT12 to ZT24) cycle. ZT is a chronobiology term, and ZT12 indicates the beginning of the dark phase under LD. Food and water were available ad libitum.

### Anesthesia

Mice were placed in a small chamber for anesthesia using 4% desflurane (0.52 MAC (minimum alveolar anesthetic concentration)^17^), which was initiated with a 4 L/min flow of 50% oxygen. After anesthesia induction, the fresh gas flow was decreased to 1 L/min. The anesthetic chamber was placed on a heated plate to maintain normal body temperature in the mice. Anesthesia was performed for 6 h^11^. These procedures were performed under dark conditions, and a dim red light that did not affect the circadian rhythm was used as needed.

### Rest/activity rhythm phase shift measurements

Seventy-two mice were used for this experiment. A commercially available mouse running wheel was used to monitor the rest/activity rhythm of mice. We set the magnetometric sensor behind the wheel embedded magnets and used a self-made device and software to count the wheel rotations. The rest/activity rhythm of mice was recorded automatically from the beginning to the end of the experiment.

**Figure 1:**
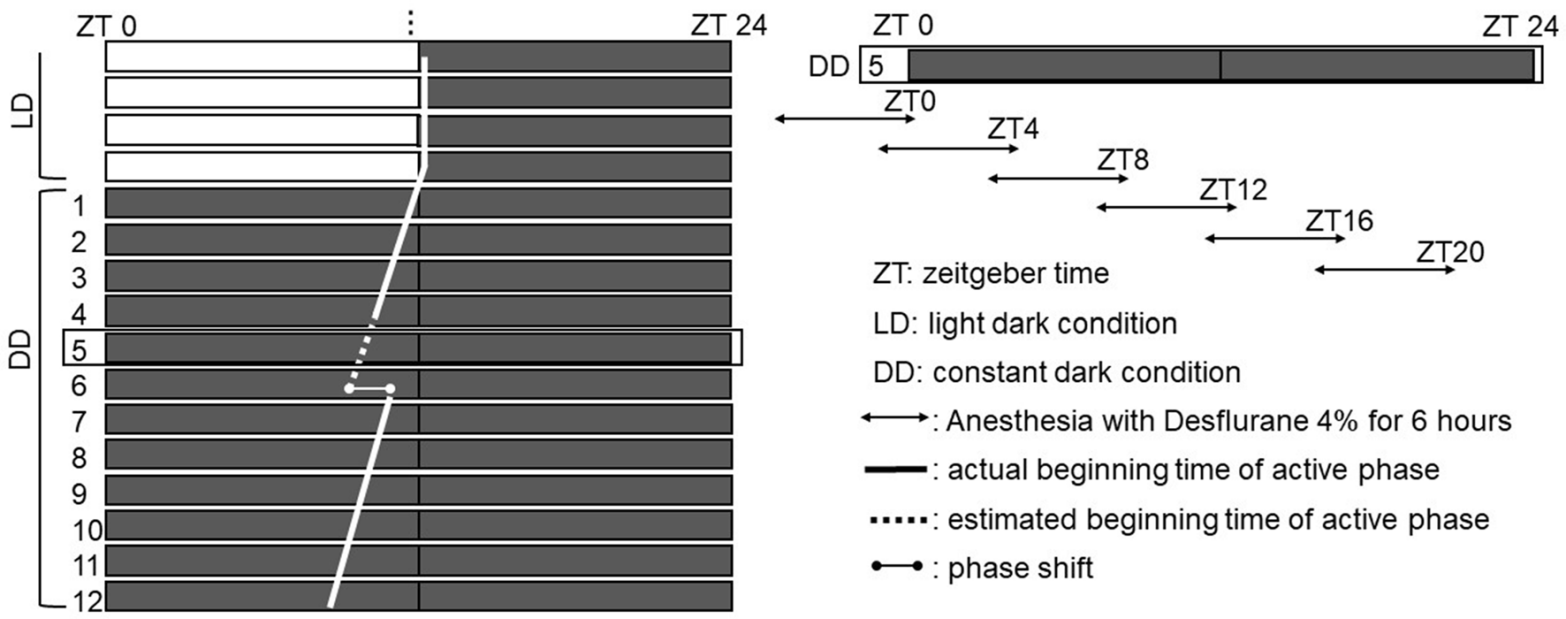
Rest/activity rhythm phase shift measurements in mice. LD started more than 10 days before the initiation of DD. The light phase was from ZT0 to 12 in LD. Anesthesia was performed on DD day 5 (left), and mice were divided into 6 subgroups according to the time-of-day of anesthesia (right; n = 6 per subgroup). In the group without anesthesia, the same procedure was performed when anesthesia was started. The time at the beginning of the active phase in mice (thick line) was determined from the actogram, and the time on DD day 6 was estimated. The difference from the estimated and actual beginning time of the active phase on DD day 6 was defined as the phase shift after anesthesia.

After adaptation with the running wheel, the dark conditions (DD; constant dark conditions) were initiated on day 1. Mice were divided into two groups (n = 36 per group). One group was anesthetized with desflurane under DD conditions on day 5. The other group was not anesthetized but was placed in the anesthetic chamber and then back into its cage. The two groups were each divided into six subgroups (ZT0, ZT4, ZT8, ZT12, ZT16, and ZT20; n = 6 per subgroup) based on the anesthetic procedure time of day (Fig. 1).

After the anesthetic procedure, the mice were placed back into their original cages. Their rest/activity rhythm was monitored for more than 6 days after the anesthetic procedure. We evaluated the phase shift, which is the difference between the actual and predicted start time of day for the active phase on the day after anesthesia (Fig. 1). We also evaluated the circadian period for each group.

### SCN sample collection

**Figure 2:**
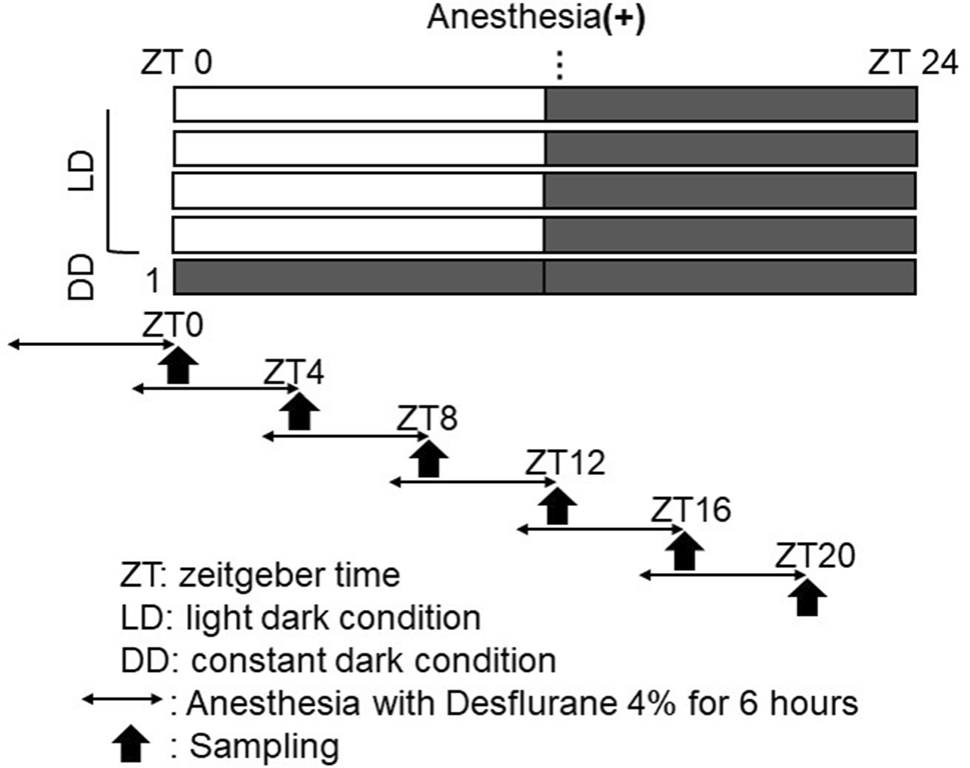
Protocol for SCN sampling in mice. After adaptation, anesthesia was performed on DD day 1. Mice were divided into 6 subgroups according to the time-of-day of anesthesia (n = 4 per subgroup). SCN sampling was performed at the end of anesthesia. In the group without anesthesia, SCN sampling was performed at the same time without anesthesia.

Samples were collected from 48 mice. After adaptation, DD conditions were initiated on day 1. Mice were divided into two groups (n = 24 per group). The anesthesia group was exposed to 4% desflurane for 6 h, and the SCN sample was collected immediately after anesthesia. The other group did not undergo desflurane anesthesia before the SCN was collected. The two groups were each divided into six subgroups based on the time of day at which the sample was collected (n = 4 per subgroup). Samples were collected at ZT0, ZT4, ZT8, ZT12, ZT16, and ZT20 on day 1 (Fig. 2). Sample collection was performed under dim red light. Mice were euthanized by cervical dislocation. The brain was quickly removed within a few minutes and placed into RNAlater (Qiagen, Hilden, Germany). The brain was immediately cut with a brain slicer (Muromachi Kikai, Tokyo, Japan) into sections (1.5 mm thick), which included the SCN. The bilateral SCN was resected as a triangular prism (~ 2 mm a side) using micro scissors in RNAlater reagent and placed into a collection tube with fresh RNAlater (150 µl). These processes required only about 15 min. The collected samples were stored at 4ºC for approximately 1 week until RNA extraction and reverse transcription.

### Quantitative real-time polymerase chain reaction (q-PCR)

Total RNA was extracted from the collected SCN samples using an RNeasy Mini Kit (Qiagen). The RNA was reverse transcribed into cDNA using a QuantiTect Reverse Transcription Kit (Qiagen). q-PCR was performed using a StepOnePlus Real-Time PCR system (Applied Biosystems, Foster, CA, USA) and QuantiTect SYBR Green PCR Kit (Qiagen). Target gene expression was quantified using the ΔΔCt method.

The target clock gene primers were as follows: *Per2* forward primer, 5′-ATCAGCCATGTTGCCGTGTC-3′ and *Per2* reverse primer, 5′-CGTGCTCAGTGGCTGCTTTC-3′; *Bmal* forward primer, 5′-ACGACATAGGACACCTCGCAGA-3′ and *Bmal* reverse primer, 5′-TCCTTGGTCCACGGGTTCA-3′; *Clock* forward primer, 5′-AGCTGCATATTGCCGTTGTAATTG-3′ and *Clock* reverse primer, 5′-CTGAGTGAAGGCATGCTGGTG-3′; and *Cry1* forward primer, 5′-GGATCCACCATTTAGCCAGACAC-3′ and *Cry1* reverse primer, 5′-CATTTATGCTCCAATCTGCATCAAG-3′.

The internal control gene primers were as follows: *Actb* forward primer, 5′-CATCCGTAAAGACCTCTATGCCAAC-3′ and *Actb* reverse primer, 5′-ATGGAGCCACCGATCCACA-3′.

The protocol for q-PCR consisted of three stages. The holding stage kept the temperature at 95 °C for 15 min; the cycling stage repeated 40 cycles of 94 °C for 15 s and then 58 °C for 1 min; and the melt curve stage was confirmed by 95 °C for 15 s and 60 °C for 1 min, followed by increasing the temperature in increments of 0.3–95 °C for 15 s.

### Statistical analysis

To determine the statistical difference in the phase shift and circadian period between desflurane-anesthetized mice and controls, a Mann–Whitney *U*-test was applied. Time-dependent fluctuations in the phase shift and relative changes in gene expression were analyzed using the Kruskal–Wallis test followed by the Mann–Whitney *U*-test, and the *p* value was adjusted by Bonferroni correction. All statistical analyses were performed using R (version 3.3.2), and *p* values < 0.05 were considered statistically significant.

## Results

### Phase shift of rest/activity rhythm after desflurane anesthesia

**Figure 3:**
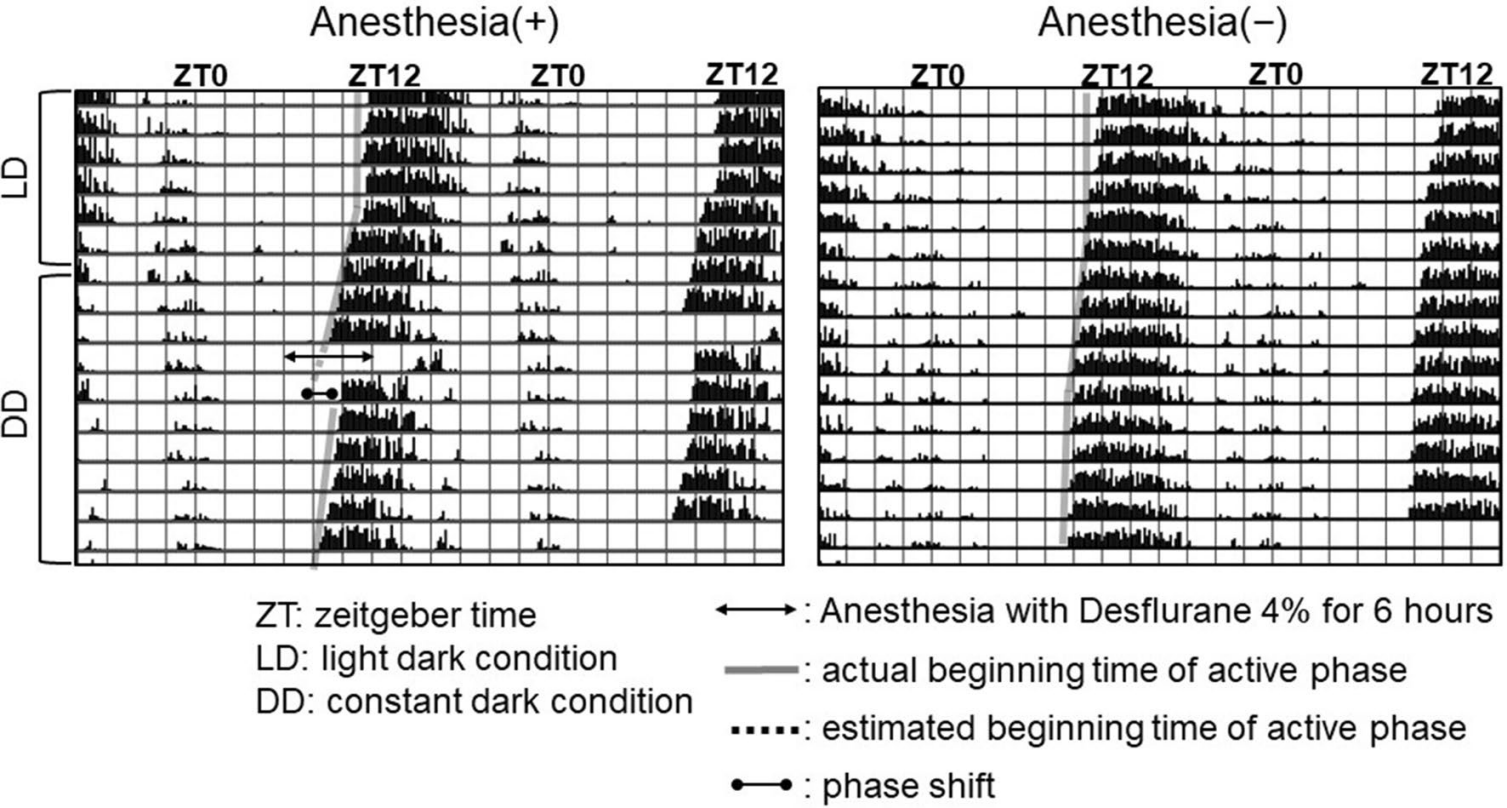
Representative double-plot actogram in mice. The number of running wheel rotations obtained from each mouse was determined in 10-min intervals to generate a bar graph in which 48 h are shown on one line. This anesthesia (+) mouse showed a phase shift of − 1.45 h, and the anesthesia (−) mouse showed no phase shift.

A total of 72 mice were used to measure the phase shift after desflurane anesthesia, and no mice were excluded during measurements. A representative double-plot actogram shows the phase delay in mice after desflurane anesthesia from ZT6 to ZT12, but there was no phase shift in the non-anesthetized mice (Fig. 3). The number of running wheel rotations obtained from each mouse was determined in 10-min intervals to generate a bar graph in which 48 h are shown in one line.

The circadian time, phase shift, and circadian period of rest/activity rhythm in each subgroup in both groups are shown in Table 1. The largest phase shift was observed in the ZT12 subgroup of the anesthesia group, and there was a significant difference between the anesthesia and non-anesthesia groups (− 1.69 h vs. − 0.58 h, *p* = 0.005). A significant difference in the phase shift was also observed between subgroups ZT4 and ZT12 (0.46 h vs. − 1.7 h, *p* = 0.032) and between subgroups ZT4 and ZT20 (0.46 h vs. − 1.1 h, *p* = 0.032) in the anesthesia group.

**Table 1:** Circadian time, phase shift, and circadian period of the rest/activity rhythm in mice. Circadian time is the subjective time of each mouse at the time-of-day of anesthesia. Phase shift (h) is the difference between the actual and predicted start time of day for the active phase on the day after anesthesia. Circadian period (h) of DD days 1–4 and DD day 6– indicate the circadian period before or after the anesthetic procedure in mice. The median (Interquartile range) of the circadian time, phase shift, and circadian period was calculated for each subgroup (n = 6). The *p* value was calculated using the Mann–Whitney *U*-test.

Group	Anesthesia (+), n = 36					
Subgroup	ZT0 (n = 6)	ZT4 (n = 6)	ZT8 (n = 6)	ZT12 (n = 6)	ZT16 (n = 6)	ZT20 (n = 6)
Circadian time	2.0 (1.7–2.3)	5.1 (5.0–6.5)	10.3 (9.5–10.4)	15.0 (14.6–15.3)	18.2 (17.9–18.6)	23.3 (21.7–23.9)
Phase shift (h)	− 0.21 (− 0.44 to − 0.10)	0.46 (0.15–0.65)*,**	− 0.71 (− 0.83 to − 0.58)	− 1.7(− 1.8 to − 1.5)*,***	− 0.75(− 1.1 to − 0.33)	− 1.1(− 1.5 to − 0.77)**
**Circadian period (h)**						
LD	23.96 (23.96–23.96)	24.00 (23.96–24.04)	24.02 (23.97–24.04)	24.02 (23.97–24.04)	23.98 (23.96–24.04)	24.00 (23.96–24.04)
DD day 1–4	23.59 (23.54–23.66)	23.77 (23.50–23.79)	23.54 (23.51–23.70)	23.40 (23.35–23.48)	23.56 (23.48–23.61)	23.33 (23.22–23.67)
DD day 6–	23.69 (23.64–23.80)	23.76 (23.62–23.82)	23.79 (23.66–23.79)*,***	23.67 (23.56–23.77)	23.62 (23.61–23.78)	23.65 (23.50–23.79)

Group	Anesthesia (−), n = 36					
Subgroup	ZT0 (n = 6)	ZT4 (n = 6)	ZT8 (n = 6)	ZT12 (n = 6)	ZT16 (n = 6)	ZT20 (n = 6)
Circadian time	2.0 (1.7–3.2)	5.7 (5.2–7.0)	10.9 (10.2–11.8)	14.4 (13.7–14.5)	18.1 (17.7–18.8)	24.3 (22.5–25.6)
Phase shift (h)	− 0.042(− 0.23 to 0.21)	− 0.33(− 0.33 to 0.23)	− 0.63(− 1.0 to − 0.17)	− 0.58(− 0.67 to − 0.37)***	− 0.21(− 0.42 to 0.19)	− 0.55(− 1.1 to − 0.30)
**Circadian period (h)**						
LD	24.04 (24.01–24.07)	23.98 (23.96–24.00)	23.98 (23.93–24.00)	23.96 (23.93–23.99)	23.98 (23.93–24.03)	23.94 (23.92–23.99)
DD day 1–4	23.60 (23.35–23.67)	23.67 (23.40–23.77)	23.42 (23.24–23.56)	23.53 (23.50–23.67)	23.58 (23.45–23.66)	23.15 (22.88–23.50)
DD day 6–	23.79 (23.69–23.90)	23.63 (23.46–23.79)	23.40 (23.22–23.51)*,***	23.46 (23.40–23.77)	23.67 (23.63–23.74)	23.62 (23.58–23.67)

Median (interquartile range).

Phase shift (h): *subgroup ZT4 versus ZT12, *p* = 0.03, **subgroupZT4 versus ZT20, *p* = 0.03, ***anesthesia (+) versus (−) in subgroupZT12, *p* = 0.005.

Circadian period DD day 6–: ****anesthesia (+) versus (−) in subgroupZT8, *p* = 0.01.

The median phase shift was delayed for − 0.67 h [interquartile range (IQR), − 1.27 to − 0.15] in the anesthesia group and − 0.33 h (IQR, − 0.67 to 0.02) in the non-anesthesia group (*p* = 0.043).

**Figure 4:**
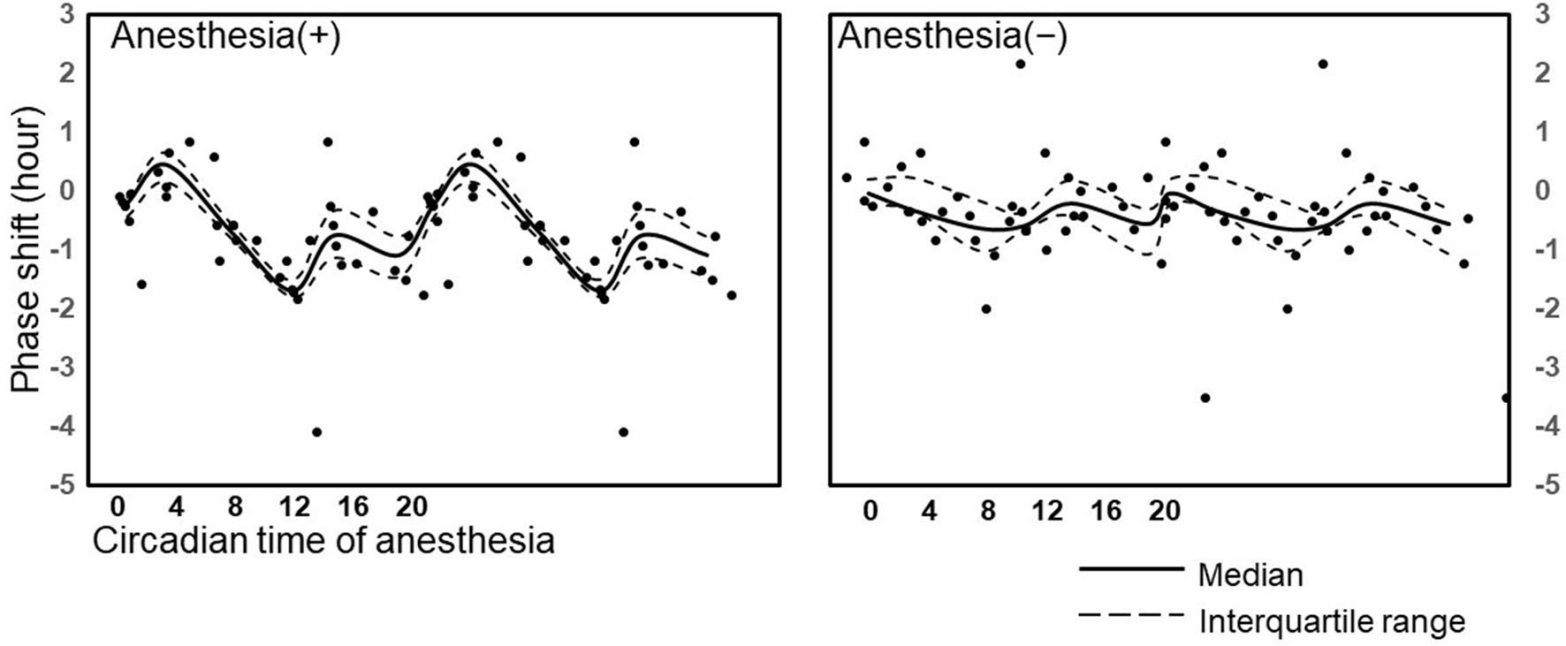
Double-plot of the phase shift based on the circadian time of anesthesia. The phase shift of all mice was plotted against the circadian time of anesthesia [left, anesthesia (+); right, anesthesia (−)].The solid line indicates the median phase shift (h), and the broken lines show the interquartile range (IQR) against the median circadian time of anesthesia in each subgroup (n = 6).

The phase shift in all mice that showed a different circadian time of the anesthetic procedure was shown in Fig. 4. Fluctuations in the extent of the phase shift were observed in mice after desflurane anesthesia (*p* = 0.00037), but these were not observed in non-anesthetized mice (*p* = 0.17; Fig. 4).

**Figure 5:**
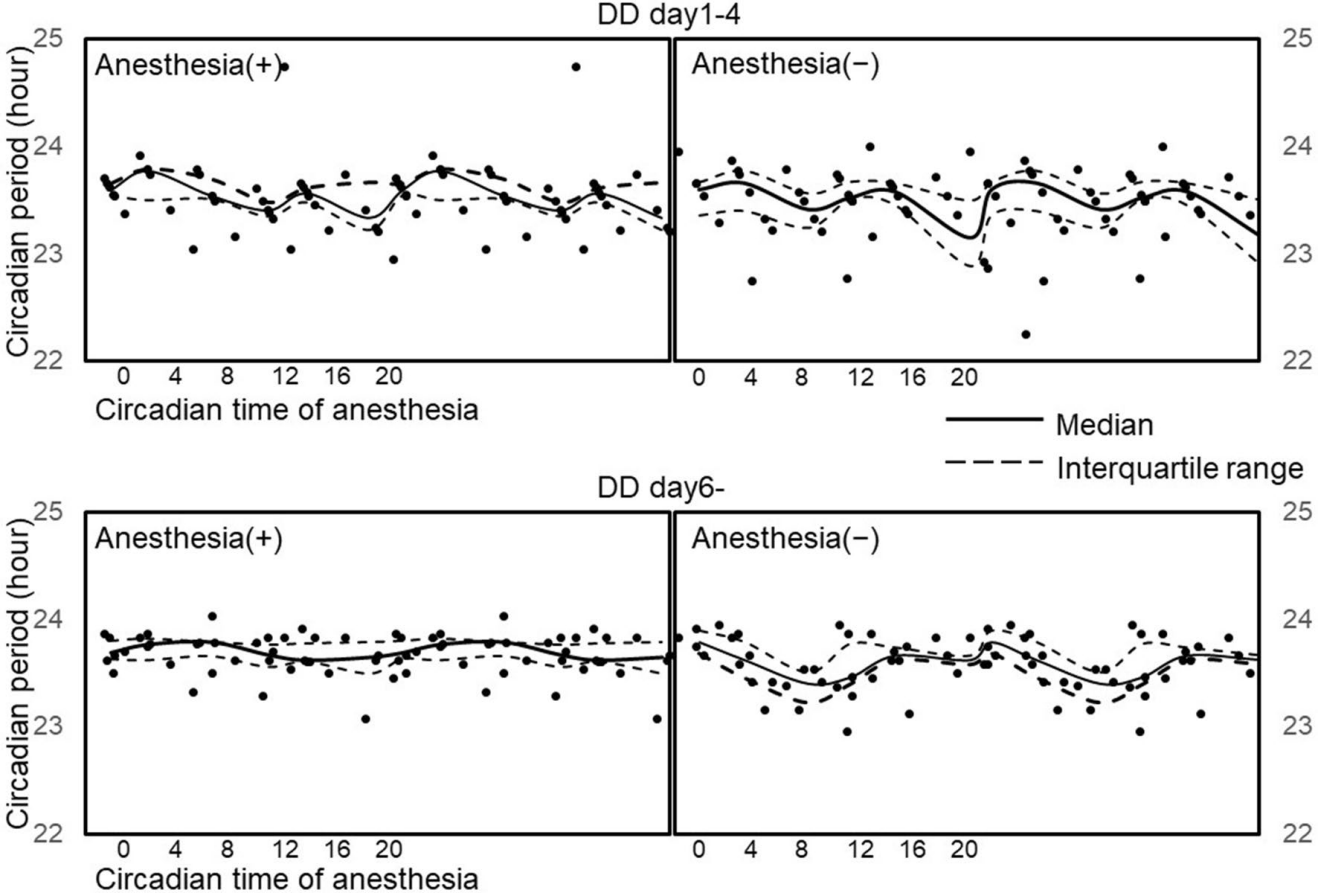
The circadian period under DD conditions in all mice. DD days 1–4 and DD day 6– indicate the DD condition before and after the anesthetic procedure, respectively. All circadian periods in the DD condition were plotted against the circadian time of the anesthetic procedure. The anesthesia (+) group is on the left, and the anesthesia (−) group is on the right. The solid and broken lines are the median and IQR of the circadian period, respectively, calculated in each subgroup.

The circadian time of the anesthetic procedure was calculated based on the circadian period before the anesthetic procedure. There was no statistically significant difference in the changes in circadian time between the anesthetized group, the non-anesthetized group, and each subgroup. The circadian period in all mice that showed a different circadian time of anesthesia was shown in Fig. 5. The circadian period was different in each mouse, but no significant difference was observed between the anesthesia and non-anesthesia groups. Within each subgroup, only ZT8 showed a significant difference in the circadian period in DD after the anesthetic procedure (*p* = 0.01) (Table 1).

### Changes in the relative expression level of clock genes after desflurane anesthesia

**Figure 6:**
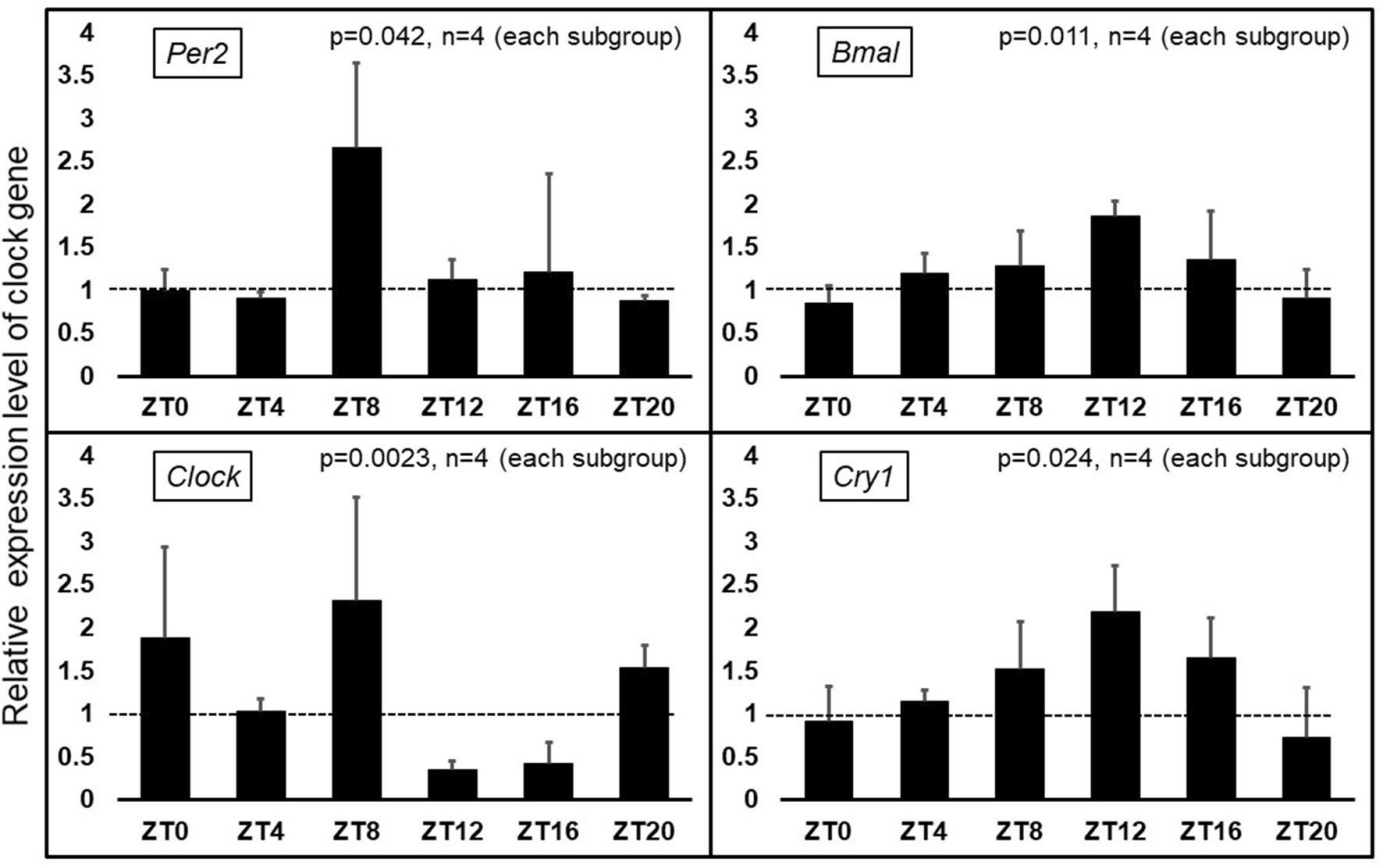
Relative mRNA expression level of clock genes in the SCN after anesthesia. The relative expression level of *Per2*, *Bmal*, *Clock,* and *Cry1* was measured as indicated in the left upper corner of each graph. The relative expression level was calculated by dividing the mRNA expression of the anesthesia group by that of the non-anesthesia group in each subgroup. The calculation was performed in groups in which the SCN was collected at the same time of day. The mRNA expression was measured by q-PCR using the ΔΔCT method. The *p* value was calculated using the Kruskal–Wallis test. Error bars indicate the standard deviation.

A total of 48 mice were used to measure the relative expression level of clock genes after desflurane anesthesia. No mice were excluded. The relative expression level of clock genes was calculated by dividing the gene expression in anesthetized mice by that of non-anesthetized mice. The calculation was performed in groups of samples that were collected at the same time of day. Therefore, the original circadian variation in clock gene expression was excluded. The relative expression level of each clock gene differed depending on the time of day of anesthesia (*Per2*: *p* = 0.042, *Bmal*: *p* = 0.011, *Clock*: *p* = 0.0023, *Cry1*: *p* = 0.024; Fig. 6). *Bmal* and *Cry1* expression levels were increased in subgroup ZT12 [*Bmal*: 1.87 (IQR, 1.95–1.82) and *Cry1*: 2.18 (IQR, 2.54–1.85)]. However, *Clock* expression was decreased after anesthesia at the same time of day [*Clock* in subgroup ZT12: 0.36 (IQR, 0.37–0.30)]. Additionally, *Per2* expression increased after anesthesia from ZT2 to ZT8 [*Per2*: 2.67 (IQR, 2.86–2.22].

## Discussion

Desflurane anesthesia shifted the rest/activity rhythm phase in mice, and the extent of the phase-shift differed depending on the time of day of anesthesia. The effects of inhaled anesthetics on the circadian rhythm have been reported for sevoflurane and isoflurane^8,9^. During the active or inactive phase, 2.5% of sevoflurane anesthesia delayed the phase of rest/activity rhythm and suppressed *Per2* expression in the mouse SCN^18^. Similarly, during the active period, 1% isoflurane anesthesia shifted the rest/activity rhythm and disturbed acetylcholine release in the rat hippocampus^9^. However, previous studies have not quantitatively investigated the influence of the time of day of anesthesia. In the present study, the phase delay was largest after anesthesia in ZT6–12, which is the latter half of the inactive phase. However, desflurane anesthesia was performed on day 5 after DD was initiated. The circadian period of mice under DD is known to be 23.28–23.77 h^19^, which is similar to our result (Table 1). Therefore, when desflurane anesthesia was performed on day 5, the beginning of the active phase was estimated to have advanced by 2.5 h. In fact, the median circadian time of subgroup ZT12 was 15.0 in the anesthesia group and 14.4 in the non-anesthesia group. Desflurane anesthesia in ZT6–12, which showed the largest phase shift, involved anesthesia administration from the late inactive phase to the early active phase subjectively in mice. Previous studies using isoflurane indicate that anesthesia at the beginning of the active phase causes a large delay in the phase shift^20^, which is consistent with our findings.

In addition, the effect of anesthesia at the beginning of the active phase on enhancing the phase delay is similar to the effect of light pulses at the beginning of the active phase that delays the circadian rhythm in nocturnal animals^21^. In mammals, light stimuli received by ipRGCs are transmitted to the SCN via the RHT^14^. Glutamate, pituitary adenylate cyclase activating polypeptide (PACAP), and aspartate are the neurotransmitters of signals from the RHT to the SCN, and glutamate plays the most important role among them. Inhaled anesthetics, including desflurane, act as an N-methyl-d-aspartate (NMDA) receptor antagonist, which competitively inhibits glutamate transmission. Blocking the input signal from the RHT to the SCN might cause a phase shift^22,23^. However, each anesthesia procedure was performed under DD in our study. Whether inhaled anesthetics time-dependently suppress the input signal from the RHT, even under dark conditions, remains unknown.

It has been reported that desflurane has different effects compared with other inhaled anesthetics on melatonin production after daytime anesthesia^13^. The sympathetic system regulates melatonin synthesis by β1 and α1 adrenergic receptor activation^24,25^. Melatonin is an endocrine hormone secreted by the pineal gland, which is under the control of the SCN circadian rhythm, and it is involved in the rest/activity rhythm of many mammals^26^. The sympathomimetic effect induced by desflurane might have influenced the original circadian rhythm through sympathetic activity^27^. Because C57BL/6 J mice do not produce melatonin, melatonin is irrelevant in the interpretation of these results. However, the sympathomimetic effect may explain why desflurane showed a significant time-dependent fluctuation in the phase shift, unlike sevoflurane.

The effects of general anesthesia on clock genes have been increasingly elucidated in recent years. Sevoflurane anesthesia suppresses *Per2* expression in the SCN, and it is related to the epigenetic regulation of clock genes^16^. Additionally, isoflurane anesthesia advances the circadian rhythm in mice, and this is related to increased *Bmal* expression^9^. We investigated the expression of four clock genes (*Per2*, *Bmal*, *Clock*, and *Cry1*) after desflurane anesthesia using real-time PCR. *Bmal* and *Cry1* showed increased expression with the peak observed after ZT6–12 anesthesia. However, *Clock* expression was most suppressed after anesthesia at the same time of day. *Per2* expression showed no significant change after ZT6–12 anesthesia, but it increased after ZT2–8 anesthesia. The SCN is the master biological clock in mammals. The rest/activity rhythm is regulated by the dorsomedial hypothalamic nucleus and the locus coeruleus, which have a circadian rhythm that is synchronized with the circadian oscillation of the SCN^28^. In our study, the phase shift in the rest/activity rhythm was enhanced after anesthesia from ZT6 to ZT12. The change in the relative expression level of clock genes was also enhanced after anesthesia at the same time of day. Clock gene expression is feedback-regulated by the levels of other clock genes and proteins. BMAL and CLOCK proteins bind to a region termed the E-box and promote the transcription of *Per2*^29,30^. The transcribed and translated PER protein binds to CRY and exerts negative feedback on its transcription^31,32^. Increased *Bmal* expression after ZT6–12 anesthesia was thought to have resulted in increased *Cry1* expression after anesthesia at the same time of day. However, *Clock* expression decreased reciprocally after ZT6–12 anesthesia compared with the increase in *Cry1* expression. Desflurane anesthesia altered the expression of each clock gene in the SCN, and this effect was dependent on the anesthesia time of the day. However, the molecular mechanisms regulating the circadian clock in the SCN are complicated, and it was difficult to determine the meaning of each alteration.

*Clock* expression was originally reported to have no circadian oscillation in the SCN. CLOCK is a histone acetyltransferase that forms a heterodimer with BMAL1 and promotes the transcription of *Per* and *Cry* mRNA via the E-box in their promoters. This is an important mechanism controlling the transcription and translation feedback loop of clock genes^33^. In fact, mice with mutant *Clock* genes show a prolonged circadian rhythm^34^. In our study, desflurane anesthesia showed a time-dependent effect on the relative mRNA expression level of *Clock*, and it was significantly decreased after ZT6–12 anesthesia. Because *Clock* shows no circadian variation in mRNA expression in the SCN, the decrease in the relative gene expression of *Clock* indicates that desflurane either directly or indirectly suppressed its expression. It is possible that decreased *Clock* expression slowed down the clock gene feedback loop and delayed the circadian rhythm phase. Further experiments are required to investigate the mechanisms by which anesthetics alter *Clock* gene expression.

In clinical practice, postoperative delirium, cognitive decline, and sleep disorders have been suggested to be associated with circadian rhythm disorders^35^. Many researchers suggest that general anesthetics may influence the development of these postoperative complications through altered clock gene expression in the SCN. Clinical applications of chronobiology have begun to be adopted in intensive care units^36^. However, the mechanisms of complications caused by the destructed circadian rhythm by anesthetics remain mostly unclarified. Based on our findings, the frequency of postoperative delirium, cognitive decline, and sleep disorders might differ depending on when the patients have surgery under inhalational anesthesia. Further clinical investigations examining the association between the time of day of anesthesia and postoperative adverse events are needed to validate this hypothesis.

There are several limitations to the present study that should be highlighted. First, anesthesia was performed using a relatively low inhaled desflurane concentration of 4%, which is about 0.52 MAC for C57BL/6 J mice^12^. Prolonged anesthesia using a high concentration of desflurane is harmful because of its respiratory irritation and circulation suppression^27^. To maintain a normal rest/activity rhythm, it was necessary to maintain anesthesia without invasive procedures to protect the airway and circulation. Thus, we must reconsider the experimental methods to understand the phase shift of mice after deeper or longer anesthesia.

In addition, in the phase shift experiment, mice were anesthetized on DD day 5. However, to quantify clock gene expression, mice were anesthetized on DD day 1. This is because each mouse has a different circadian period. Clock gene expression varies because of the individual circadian rhythms, even when we collected SCN samples at the same time on DD day 5. The individual difference in circadian rhythms at the time of SCN collection was minimized by performing anesthesia on DD day 1. Therefore, the fluctuation in clock gene expression caused by desflurane anesthesia could be measured.

Finally, mice were anesthetized with 4% desflurane for 6 h. In previous studies, the duration of anesthesia was different and ranged from 30 min to 8 h. Anesthesia for 6 h was relatively long and might mitigate the difference between subgroups. However, the amplitude of phase response curves yields the maximum for a 9-h light pulse^37^. Therefore, the anesthesia for 6-h might not have been too long to mitigate the difference of subgroups.

In conclusion, the circadian rhythm measured by the rest/activity rhythm of mice showed a phase shift after desflurane anesthesia, and the extent differed depending on the time of day of anesthesia. The phase shift was largest in the latter half of the inactive phase. Desflurane anesthesia increased the mRNA expression of four major clock genes in the SCN, and these alterations after anesthesia also differed depending on the time of day of anesthesia. Although further clinical investigations are needed, our findings might suggest that the time at which patients have surgery under inhalational anesthesia is an important factor to maintain the circadian rhythm, even when using desflurane, which enables fast-track anesthesia in clinical practice.

## References

[1] Klein DC, Moore RY, Reppert SM (1991). Suprachiasmatic Nucleus: the Mind’s Clock.

[2] Brainard, J., Gobel, M., Scott, B., Koeppen, M. & Eckle, T. Health implications of disrupted circadian rhtythms and the potential for daylight as therapy. *Anesthesiology* **122**, 1170–1175 (2015). 10.1097/ALN.0000000000000596 PMC4632990 25635592

[3] Smolensky, M. H., Hermida, R. C., Reinberg, A., Sackett-Lundeen, L. & Portaluppi, F. Circadian disruption: new clinical perspective of disease pathology and basis for chronotherapeutic intervention. *Chronobiol. Int.* **33**, 1101–1119 (2016). 10.1080/07420528.2016.1184678 27308960

[4] Dispersyn, G., Pain, L., Challet, E. & Touitou, Y. General anesthetics effects on circadian temporal structure: an update. *Chronobiol. Int.* **25**, 835–850 (2008). 10.1080/07420520802551386 19005891

[5] Simons, K. S. *et al.* Dynamic light application therapy to reduce the incidence and duration of delirium in intensive-care patients: a randomised controlled trial. *Lancet Respir. Med.* **4**, 194–202 (2016). 10.1016/S2213-2600(16)00025-4 26895652

[6] Ruggiero C (2017). Early post-surgical cognitive dysfunction is a risk factor for mortality among hip fracture hospitalized older persons. Osteoporos Int..

[7] Moskowitz, E. E. *et al.* Post-operative delirium is associated with increased 5-year mortality. *Am. J. Surg.* **214**, 1036–1038 (2017). 10.1016/j.amjsurg.2017.08.034 28947274

[8] Ohe, Y., Iijima, N., Kadota, K., Sakamoto, A. & Ozawa, H. The general anesthetic sevoflurane affects the expression of clock gene mPer2 accompanying the change of NAD+ level in the suprachiasmatic nucleus of mice. *Neurosci. Lett.* **490**, 231–236 (2011). 10.1016/j.neulet.2010.12.059 21195744

[9] Kikuchi, T. *et al.* Effects of volatile anesthetics on the circadian rhythms of rat hippocampal acetylcholine release and locomotor activity. *Neuroscience* **237**, 151–160 (2013). 10.1016/j.neuroscience.2013.01.062 23396087

[10] Gökmen, N. *et al.* Day-time isoflurane administration suppresses circadian gene expressions in both the brain and a peripheral organ, liver. *Turk. J. Anaesthesiol. Reanim.* **45**, 197–202 (2017). 10.5152/TJAR.2017.68466 PMC5579212 28868166

[11] Poulsen, R. C., Warman, G. R., Sleigh, J., Ludin, N. M. & Cheeseman, J. F. How does general anaesthesia affect the circadian clock?. *Sleep Med. Rev.* **37**, 35–44 (2018). 10.1016/j.smrv.2016.12.002 28162920

[12] Ocmen, E. *et al.* Effect of day/night administration of three different inhalational anesthetics on melatonin levels in rats. *Kaohsiung J. Med. Sci.* **32**, 302–305 (2016). 10.1016/j.kjms.2016.04.016 PMC11916371 27377842

[13] Detwiler, P. B. Phototransduction in retinal ganglion cells. *Yale J. Biol. Med.* **91**, 49–52 (2018). PMC5872641 29599657

[14] Mistlberger RE, Skene DJ (2005). Nonphotic entrainment in humans?. J. Biol. Rhythms..

[15] Mori, K. *et al.* Epigenetic suppression of mouse per2 expression in the suprachiasmatic nucleus by the inhalational anesthetic, sevoflurane. *PLoS ONE* **9**, e87319. 10.1371/journal.pone.0087319 (2014). 10.1371/journal.pone.0087319 PMC3909093 24498074

[16] Wang D (2020). Circadian differences in emergence from volatile anaesthesia in mice: involvement of the locus coeruleus noradrenergic system. Br. J. Anaesth..

[17] JSonner, J. M., Gong, D. & Eger, E. I. Naturally occurring variability in anesthetic potency among inbred mouse strains. *Anesth. Analg.* **91**, 720–726 (2000). 10.1097/00000539-200009000-00042 10960407

[18] Kadota, K. *et al.* Time-dependent repression of mPer2 expression in the suprachiasmatic nucleus by inhalation anesthesia with sevoflurane. *Neurosci. Lett.* **528**, 153–158 (2012). 10.1016/j.neulet.2012.07.061 22902991

[19] Schwartz WJ, Zimmerman P (1990). Circadian timekeeping in BALB/c and C57BL/6 inbred mouse strains. J. Neurosci..

[20] Ludin, N. M., Cheeseman, J. F., Merry, A. F., Millar, C. D. & Warman, G. R. The effects of the general anaesthetic isoflurane on the honey bee (Apis mellifera) circadian clock. *Chronobiol. Int.* **33**, 128–133 (2016). 10.3109/07420528.2015.1113987 26730506

[21] Pittendrigh CS, Aschoff J (1981). Circadian systems: general perspective. Handbook of Behavioral Neurobiology.

[22] Campagna, J. A., Miller, K. W. & Forman, S. A. Mechanisms of actions of inhaled anesthetics. *N. Engl. J. Med.* **348**, 2110–2124 (2003). 10.1056/NEJMra021261 12761368

[23] Ohi, K., Takashima, M., Nishikawa, T. & Takahashi, K. N-Methyl-d-Aspartate receptor participates in neuronal transmission of photic information through the retinohypothalamic tract. *Neuroendocrinology* **53**, 344–348 (1991). 10.1159/000125740 1675438

[24] Naguib, M., Gottumukkala, V. & Goldstein, P. A. Melatonin and anesthesia: a clinical perspective. *J. Pineal. Res.* **42**, 12–21 (2007). 10.1111/j.1600-079X.2006.00384.x 17198534

[25] Bellapart J, Boots R (2012). Potential use of melatonin in sleep and delirium in the critically ill. Br. J. Anaesth..

[26] Claustrat, B., Brun, J. & Chazot, G. The basic physiology and pathophysiology of melatonin. *Sleep Med. Rev.* **9**, 11–24 (2005). 10.1016/j.smrv.2004.08.001 15649735

[27] Ebert, T. J. & Stowe, D. F. Neural and endothelial control of the peripheral circulation: implications for anesthesia—part I, neural control of the peripheral vasculature. *J. Cardiothorac. Vasc. Anesth.* **10**, 147–158 (1996). 10.1016/s1053-0770(96)80190-x 8634380

[28] Aston-Jones, G., Chen, S., Zhu, Y. & Oshinsky, M. L. A neural circuit for circadian regulation of arousal. *Nat. Neurosci.* **4**, 732–738 (2001). 10.1038/89522 11426230

[29] DeBruyne, J. P., Weaver, D. R. & Reppert, S. M. CLOCK and NPAS2 have overlapping roles in the suprachiasmatic circadian clock. *Nat. Neurosci.* **10**, 543–545 (2007). 10.1038/nn1884 PMC2782643 17417633

[30] Gekakis, N. *et al.* Role of the CLOCK protein in the mammalian circadian mechanism. *Science* **280**, 1564–1569 (1998). 10.1126/science.280.5369.1564 9616112

[31] Wijnen, H. & Young, M. W. Interplay of circadian clocks and metabolic rhythms. *Annu. Rev. Genet.* **40**, 409–448 (2006). 10.1146/annurev.genet.40.110405.090603 17094740

[32] Yu, W., Nomura, M. & Ikeda, M. Interactivating feedback loops within the mammalian clock: BMAL1 is negatively autoregulated and upregulated by CRY1, CRY2, and PER2. *Biochem. Biophys. Res. Commun.* **290**, 933–941 (2002). 10.1006/bbrc.2001.6300 11798163

[33] Doi, M., Hirayama, J. & Sassone-Corsi, P. Circadian regulator CLOCK is a histone acetyltransferase. *Cell* **125**, 497–508 (2006). 10.1016/j.cell.2006.03.033 16678094

[34] Vitaterna, M. H. *et al.* Mutagenesis and mapping of a mouse gene, clock, essential for circadian behavior. *Science* **264**, 719–725 (1994). 10.1126/science.8171325 PMC3839659 8171325

[35] Riemersma-van der Lek, R. F. *et al.* Effect of bright light and melatonin on cognitive and noncognitive function in elderly residents of group care facilities. *JAMA* **299**, 2642–2655 (2008). 10.1001/jama.299.22.2642 18544724

[36] Pulak, L. M. & Jensen, L. Sleep in the intensive care unit: a review. *J. Intensive Care Med.* **31**, 14–23 (2014). 10.1177/0885066614538749 24916753

[37] Comas M, Beersma DG, Spoelstra K, Daan S (2006). Phase and period responses of the circadian system of mice (*Mus musculus*) to light stimuli of different duration. J. Biol. Rhythms.

