# Author Correction: Desflurane anesthesia shifts the circadian rhythm phase depending on the time of day of anesthesia

**DOI:** 10.1038/s41598-021-84378-4

**Published:** 2021-03-09

**Authors:** Ryo Imai, Hiroshi Makino, Takasumi Katoh, Tetsuro Kimura, Tadayoshi Kurita, Kazuya Hokamura, Kazuo Umemura, Yoshiki Nakajima

**Affiliations:** 1 Department of Anesthesiology, Hamamatsu University School of Medicine, 1-20-1 Handayama, Hamamatsu, Shizuoka 431-3192 Japan; 2 Department of Medical Education, Hamamatsu University School of Medicine, Hamamatsu, Japan; 3 Department of Pharmacology, Hamamatsu University School of Medicine, Hamamatsu, Japan

Correction to: *Scientific Reports* 10.1038/s41598-020-75434-6, published online 26 October 2020

The original version of this Article contained errors in the Reference list. In references 2–5, 7–15, and 17–36 the author first and last names were reversed.

These errors have now been corrected in the HTML and PDF versions of the Article.

